# l-Lactic acid production from fructose by chitosan film-coated sodium alginate-polyvinyl alcohol immobilized *Lactobacillus pentosus* cells and its kinetic analysis

**DOI:** 10.1186/s40643-021-00380-8

**Published:** 2021-04-07

**Authors:** Jianfei Wang, Huanyu Guo, Jiaqi Huang, Shaoming Jiang, Shibo Hou, Xingyu Chen, Hujie Lv, Xudong Bi, Maolin Hou, Hebei Lin, Yuming Lu, Jinyue Qiao, Ruiyi Yang, Shijie Liu

**Affiliations:** 1 Department of Chemical Engineering, SUNY College of Environmental Science and Forestry, Syracuse, NY13210 USA; 2 The Center for Biotechnology and Interdisciplinary Studies (CBIS) at Rensselaer Polytechnic Institute, Troy, NY12180 USA; 3 California State University, Los Angeles (CSULA), Los Angeles, CA 90032 USA; 4 Department of Biomedical Engineering, City University of Hong Kong, Hong Kong, 999077 China

**Keywords:** Cell immobilization, Kinetics, Optimization, Batch fermentation, Lactic acid

## Abstract

Under the optimal conditions of immobilization and fermentation, the highest LA yield of 0.966 ± 0.006 g/g fructose and production rate of 2.426 ± 0.018 g/(L × h) with an error of -0.5% and -0.2% to the predicted results were obtained from batch fermentation by the CS film-coated SA-PVA immobilized *L. pentosus* cells. The LA yield and production rate of these immobilized cells were 2.7% and 10.1% higher than that of normal SA-PVA immobilized cells respectively, and they were 5.7% and 48.4% higher than that of free cells, respectively. The effect of temperature on different types of immobilized cells and free cells was significantly different, but the effect of pH on different types of cells was not much different. The kinetic models could effectively describe the different fermentation performances of three types of cells. The immobilized cells have excellent reusability to conduct 9 runs of repeated batch fermentation. 
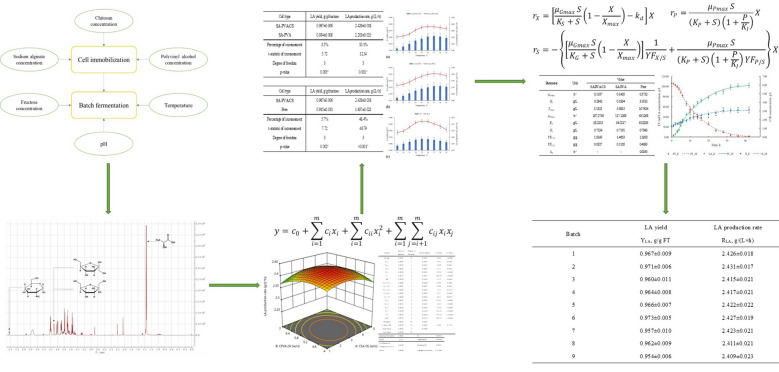

## Introduction

Lactic acid (LA) is considered to be an important precursor for the synthesis of some oxygen-containing compounds and other chemical intermediates (Olszewska-Widdrat et al. 2019). In recent years, polylactic acid (PLA) has received extensive attention due to its biocompatibility and biodegradability, which has led to a further increase in the demand and production of LA (Agarwal et al. 1998; Abd Alsaheb et al. 2015). LA can be obtained through chemical synthesis or fermentation (Abdel-Rahman et al. 2011). Compared with the chemical synthesis that produces DL-LA racemic mixture as a by-product, fermentation by lactic acid bacteria (LAB) can produce L-LA or D-LA with high optical purity, thereby further reducing the cost of separation and purification of LA products (Zhao et al. 2016; Ricci et al. 2019).

The *Lactobacillus pentosus* (*L. pentosus*) is a suitable LAB to synthesize L-LA from different carbon sources by different fermentation types (Bustos et al. 2005). Generally, *L. pentosus* produces L-LA from hexose by homologous fermentation via the Embden–Meyerhoff–Parnas (EMP) pathway (Eq. (1)) or from pentoses by heterologous fermentation via the pentose phosphoketolase (PK) pathway (Eq. (2)) (Martinez et al. 2013; Mayo et al. 2010). However, *L. pentosus* can also synthesis L-LA from hexose by heterologous fermentation via the pentose phosphoketolase (PK) pathway (Eq. (3)) (Gao et al. 2011). The theoretical yields are 1 g/g hexose, 0.6 g/g pentosus, and 0.5 g/g hexose, respectively.

1 $$ {\text{C}}_{{6}} {\text{H}}_{{{12}}} {\text{O}}_{{6}} \to {\text{2 C}}_{{3}} {\text{H}}_{{6}} {\text{O}}_{{3}} $$

2 $$ {\text{C}}_{{5}} {\text{H}}_{{{10}}} {\text{O}}_{{5}} \to {\text{ C}}_{{3}} {\text{H}}_{{6}} {\text{O}}_{{3}} {\text{ + CH}}_{{3}} {\text{COOH}} $$

3 $$ {\text{C}}_{{6}} {\text{H}}_{{{12}}} {\text{O}}_{{6}} \to {\text{ C}}_{{3}} {\text{H}}_{{6}} {\text{O}}_{{3}} {\text{ + C}}_{{2}} {\text{H}}_{{5}} {\text{OH + CO}}_{{2}} $$

Currently, cell immobilization technology is still a research hotspot. Cells immobilized by suitable materials have higher initial cell density and metabolic activity, thereby performing higher product yield and production rate (Kumar et al. 2014). Gel encapsulation is a common method of cell immobilization. During the cell immobilization process, the gel material reacts with the cross-linking agent to encapsulate the cells in the beads (Tang et al. 2017). Among various materials, sodium alginate (SA), polyvinyl alcohol (PVA), and chitosan (CS) have good properties and relatively low prices, which are ideal gel materials for cell immobilization. At present, the related studies still focus more on the immobilization by the single gel material. However, a single gel material is not suitable for all strains and fermentation conditions due to the specific properties of this material. The immobilized cell beads prepared from the mixture of two gel materials have also been studied, and the more common ones are SA-PVA beads and SA-CS beads. The SA-PVA bead has good surface properties and mechanical strength (Wang, Huang, Laffend et al. 2020a, b, c). However, the cell release from this type of beads cannot be effectively controlled. The SA-CS bead can effectively avoid cell release, but it has low mechanical strength and stability, resulting in a limited range of its applications due to the fermentation conditions. Jeon et al. (2019) studied the mechanical stability of both SA-CS beads and SA-PVA beads. They reported that SA-CS beads disintegrated at 1500 rpm centrifugation, while SA-PVA beads could tolerate centrifugal speeds below 2000 rpm. Dong et al. (2017) also reported that SA-PVA beads have higher mechanical strength and activity recovery than SA-CS beads. Therefore, the SA-PVA immobilized cells coated with a CS film were used in the study, which could advantage of each material and avoiding the decrease in mass transfer efficiency and other properties of each material caused by the traditional immobilization method of mixing three materials as one gel mixture. In addition, fermentation conditions including the concentration of each gel material for preparing immobilized cell beads need to be considered to ensure the mass transfer efficiency of the beads and the metabolic activity of the cells. At present, the kinetic analysis of the fermentation by immobilized cells is still very limited. Therefore, establishing a suitable kinetic model can accurately and effectively describe the performance of immobilized cells.

In this study, the CS film-coated SA-PVA immobilized *L. pentosus* cells were used for L-LA production from fructose (FT) by batch fermentation. The conditions of immobilization and fermentation were optimized by Box–Behnken design. The effects of temperature and pH on the fermentation performance of CS film-coated SA-PVA immobilized cells, normal SA-PVA immobilized cells, and free cells were compared. The kinetics of cell growth, L-LA synthesis, and fructose consumption of three types of cells were also studied and compared. The performance of CS film-coated SA-PVA immobilized cells in repeated batch fermentation was discussed.

## Materials and methods

### Seed culture preparation

The freeze-dried *L. pentosus* ATCC 8041 strain obtained from the American Type Culture Collection (ATCC) was activated in de Man, Rogosa and Sharpe (MRS) medium for 20 h on a rotary shaker before batch fermentation. The temperature and shaking speed were controlled at 37 °C and 150 rpm, respectively.

### Box–Behnken design

Box–Behnken design was applied to optimize six selected parameters that were found to have significant effects in preliminary experiments based on Plackett–Burman design (Table 1). Single-factor preliminary experiments were applied to determine the range of each parameter for the highest LA yield and production rate, which can be selected for Box–Behnken design (data not shown).

**Table 1:** The range of variables for L-LA fermentation of CS film-coated SA-PVA immobilized *L. pentosus* cells from FT

Factor	Variable	Code	Unit	Level		
				−1	0	+1
1	Sodium alginate concentration	C_SA_	% (w/v)	1	3	5
2	Polyvinyl alcohol concentration	C_PVA_	% (w/v)	4.0	5.5	7.0
3	Chitosan concentration	C_CS_	% (w/v)	0.2	0.5	0.8
4	Fructose concentration	C_FT_	g/L	90	105	120
5	Temperature	T	°C	31	35	39
6	pH	pH		5	6	7

### Cell immobilization

The SA and PVA were dissolved in sterile deionized water at 30 °C and 80 °C, respectively. Two gel solutions were then mixed for SA-PVA hydrogel with the specific concentration of each material. The concentrated seed culture with a volume of 5 ml and a cell density of 3.08 × 10^8^ CFU/ml (8.49 log CFU/ml) was injected into 100 ml SA-PVA hydrogel solution and fully mixed by continuous stirring. The hydrogel solution containing cells was injected into the mixed cross-linking agent solution of 0.1 M CaCl_2_ and 2.5% H_3_BO_3_ by a syringe to prepare immobilized cell beads with the diameter of 2.2 ± 0.5 mm (Wang et al. 2020a, b, c). The cross-linking process was conducted at 4 °C in a refrigerator for 4 h. The CS solution with specific concentration was prepared by dissolving CS into the glacial acetic acid solution and adding 1 M NaOH solution to adjust pH to 5.6–6.0 (Zhou et al. 1998). The SA-PVA immobilized cell beads were washed by sterile deionized water and were subsequently immersed into the CS solution and stirred moderately on a shaker for 1 h. The CS film-coated beads were then immersed into 0.3% glutaraldehyde solution and stirred moderately for 20 min at pH 5.4 controlled by phosphate buffer. The concentration and treatment time of glutaraldehyde need to be strictly controlled to avoid damage to cell viability (Xu et al. 2012; Gür et al. 2018). The prepared CS film-coated SA-PVA immobilized cells were washed by sterile deionized water and stored in the peptone solution at 4 °C. Before batch fermentation, the immobilized cells were activated in MRS medium for 8 h.

### Batch fermentation

The 1.0 L New Brunswick Bioreactor was used for batch fermentation with a working volume of 800 ml. The components in the fermentation medium are FT with a specific concentration, 4 g/L yeast extract, 2 g/L K_2_HPO_4_, 2 g/L KH_2_PO_4_, and 0.78 g/L MgSO_4_. The fermentation temperature and pH were maintained at specific levels by the control system of the bioreactor. The stirring speed was controlled at 100 rpm by a magnetic stirrer to avoid bead breakage caused by impellers. The airflow rate was maintained at 20 ml/min. Repeated batch fermentation was carried out under optimized factors, while other conditions remain the same.

### Determination of cell concentration

The 0.2 M sodium citrate solution was used to dissolve beads for cell recycle. The weight concentration of encapsulated cells and released cells was determined based on the optical density (OD) at the wavelength of 600 nm. The linear calibration curve was generated based on the linear relationship between OD values and standard cell solutions with a dry cell weight concentration. The trendline equation of this linear calibration curve was applied for the calculation of cell concentrations based on the corresponding OD values (Wang et al. 2020a, b, c). The solution of encapsulated cells with a dilution rate of 10^–9^ was cultivated on the MRS agar for the calculation of cell density in beads.

### Determination of fructose and LA concentrations

The concentrations of fructose and LA were determined by proton nuclear magnetic resonance (^1^H NMR) spectroscopy. The NMR samples were mixed with 0.5 ml supernatant of the centrifuged fermentation broth, 0.1 ml internal standard, and 0.4 ml deuterium oxide in 5-mm-o.d. NMR tubes. The internal standard consisted of 0.1% wt trimethylsilyl propionate, 0.2% wt trimethylamine, 4.2% wt glucosamine, and 95.5% wt deuterium oxide (Buyondo and Liu 2013). The MestReNova software was applied to integrate the peak areas of fructose and LA on the NMR spectrum. The trendline equation of the linear calibration curve generated based on the peak areas and standard solutions of substance concentrations were applied to calculate the concentrations of fructose and LA.

### Statistical analysis

The Design Expert (Version 11) software was applied for experimental design and parametric optimization (Table 2). The Minitab software was applied for the calculation of t-statistics. The effects of all factors and their interactions on LA yield and LA production rate were described by response surface methodology (RSM). For box-Behnken design, a quadratic model would be generated as Eq. (4):

4 $$ y = c_{0} + \mathop \sum \limits_{i = 1}^{m} c_{i} x_{i} + \mathop \sum \limits_{i = 1}^{m} c_{ii} x_{i}^{2} + \mathop \sum \limits_{i = 1}^{m} \mathop \sum \limits_{j = i + 1}^{m} c_{ij} x_{i} x_{j} , $$

**Table 2:** The LA yield and LA production rate of CS film-coated SA-PVA immobilized *L. pentosus* cells obtained under different treatments in batch fermentation from FT

Run	Factor						Response	
	Sodium alginate concentration C_SA_, % (w/v)	Polyvinyl alcohol concentration C_PVA_, % (w/v)	Chitosan concentration C_CS_, % (w/v)	Fructose concentration C_FT_, g/L	Temperature T, °C	pH	LA yield Y_LA_, g/g FT	LA production rate R_LA_, g/(L × h)
1	3	5.5	0.2	90	35	7	0.954 ± 0.010	2.338 ± 0.018
2	3	7.0	0.5	105	39	7	0.975 ± 0.005	2.362 ± 0.024
3	1	5.5	0.2	105	35	5	0.950 ± 0.006	2.340 ± 0.027
4	5	5.5	0.2	105	35	5	0.959 ± 0.008	2.345 ± 0.023
5	1	7.0	0.5	90	35	6	0.955 ± 0.011	2.322 ± 0.022
6	5	7.0	0.5	120	35	6	0.968 ± 0.005	2.368 ± 0.024
7	3	5.5	0.5	105	35	6	0.966 ± 0.007	2.420 ± 0.019
8	3	5.5	0.5	105	35	6	0.969 ± 0.006	2.423 ± 0.021
9	3	5.5	0.2	120	35	5	0.954 ± 0.009	2.327 ± 0.025
10	1	5.5	0.5	120	31	6	0.958 ± 0.009	2.331 ± 0.027
11	3	4.0	0.2	105	31	6	0.948 ± 0.008	2.336 ± 0.024
12	5	5.5	0.5	120	39	6	0.970 ± 0.006	2.375 ± 0.020
13	5	5.5	0.8	105	35	7	0.976 ± 0.005	2.330 ± 0.023
14	5	5.5	0.5	90	31	6	0.956 ± 0.011	2.314 ± 0.028
15	3	7.0	0.5	105	31	5	0.956 ± 0.009	2.299 ± 0.029
16	5	4.0	0.5	120	35	6	0.968 ± 0.007	2.356 ± 0.025
17	1	5.5	0.8	105	35	5	0.949 ± 0.009	2.327 ± 0.024
18	3	7.0	0.8	105	39	6	0.968 ± 0.006	2.357 ± 0.022
19	1	5.5	0.5	90	39	6	0.952 ± 0.009	2.342 ± 0.021
20	3	4.0	0.8	105	39	6	0.968 ± 0.007	2.370 ± 0.022
21	1	5.5	0.2	105	35	7	0.957 ± 0.008	2.356 ± 0.027
22	5	5.5	0.5	90	39	6	0.959 ± 0.009	2.331 ± 0.024
23	3	5.5	0.2	120	35	7	0.967 ± 0.006	2.343 ± 0.028
24	3	4.0	0.2	105	39	6	0.956 ± 0.010	2.382 ± 0.021
25	1	7.0	0.5	120	35	6	0.968 ± 0.006	2.333 ± 0.024
26	3	4.0	0.5	105	39	7	0.963 ± 0.005	2.375 ± 0.024
27	3	5.5	0.8	120	35	7	0.967 ± 0.006	2.341 ± 0.026
28	3	4.0	0.8	105	31	6	0.961 ± 0.007	2.341 ± 0.020
29	1	5.5	0.5	90	31	6	0.949 ± 0.010	2.325 ± 0.028
30	5	5.5	0.8	105	35	5	0.964 ± 0.009	2.333 ± 0.027
31	3	5.5	0.5	105	35	6	0.970 ± 0.005	2.420 ± 0.019
32	3	7.0	0.2	105	39	6	0.961 ± 0.008	2.370 ± 0.021
33	5	5.5	0.2	105	35	7	0.959 ± 0.009	2.343 ± 0.028
34	3	5.5	0.5	105	35	6	0.974 ± 0.005	2.435 ± 0.022
35	3	7.0	0.5	105	31	7	0.958 ± 0.008	2.315 ± 0.027
36	1	5.5	0.5	120	39	6	0.969 ± 0.005	2.372 ± 0.026
37	3	4.0	0.5	105	39	5	0.953 ± 0.011	2.341 ± 0.026
38	3	5.5	0.8	90	35	7	0.961 ± 0.007	2.332 ± 0.023
39	5	7.0	0.5	90	35	6	0.961 ± 0.007	2.306 ± 0.027
40	3	7.0	0.2	105	31	6	0.960 ± 0.006	2.341 ± 0.024
41	3	5.5	0.2	90	35	5	0.947 ± 0.011	2.322 ± 0.024
42	3	4.0	0.5	105	31	5	0.950 ± 0.008	2.329 ± 0.021
43	5	4.0	0.5	90	35	6	0.955 ± 0.009	2.320 ± 0.029
44	3	5.5	0.5	105	35	6	0.970 ± 0.006	2.426 ± 0.020
45	1	5.5	0.8	105	35	7	0.960 ± 0.007	2.343 ± 0.021
46	3	5.5	0.8	120	35	5	0.961 ± 0.008	2.343 ± 0.023
47	3	4.0	0.5	105	31	7	0.952 ± 0.009	2.327 ± 0.028
48	3	7.0	0.8	105	31	6	0.967 ± 0.006	2.328 ± 0.025
49	3	5.5	0.8	90	35	5	0.954 ± 0.009	2.298 ± 0.026
50	1	4.0	0.5	90	35	6	0.949 ± 0.010	2.378 ± 0.022
51	3	5.5	0.5	105	35	6	0.964 ± 0.008	2.426 ± 0.023
52	1	4.0	0.5	120	35	6	0.962 ± 0.007	2.366 ± 0.027
53	5	5.5	0.5	120	31	6	0.958 ± 0.010	2.316 ± 0.020
54	3	7.0	0.5	105	39	5	0.959 ± 0.009	2.346 ± 0.018

where $y$, ${x}_{i}$, and ${x}_{j}$ represent predicted responses, $i$ th factor, and $j$ th factor, respectively. Where as ${c}_{i}$, ${c}_{ii}$, and ${c}_{ij}$ represent coefficients of linear terms, quadratic terms, and interaction terms, respectively. The ${c}_{0}$ includes the offset constant and the random error (Agrawal et al. 2020).

### Kinetic analysis

The ODEXLIMS function developed in Excel was applied to verify the adequacy of kinetic models and calculate the kinetic parameters by simultaneously solving the equations (Liu 2020). The Excel solver was applied to modify the kinetic parameters by minimizing the variance between the experimental data and predicted values.

## Results and discussions

### Result of NMR analysis

**Fig. 1:**
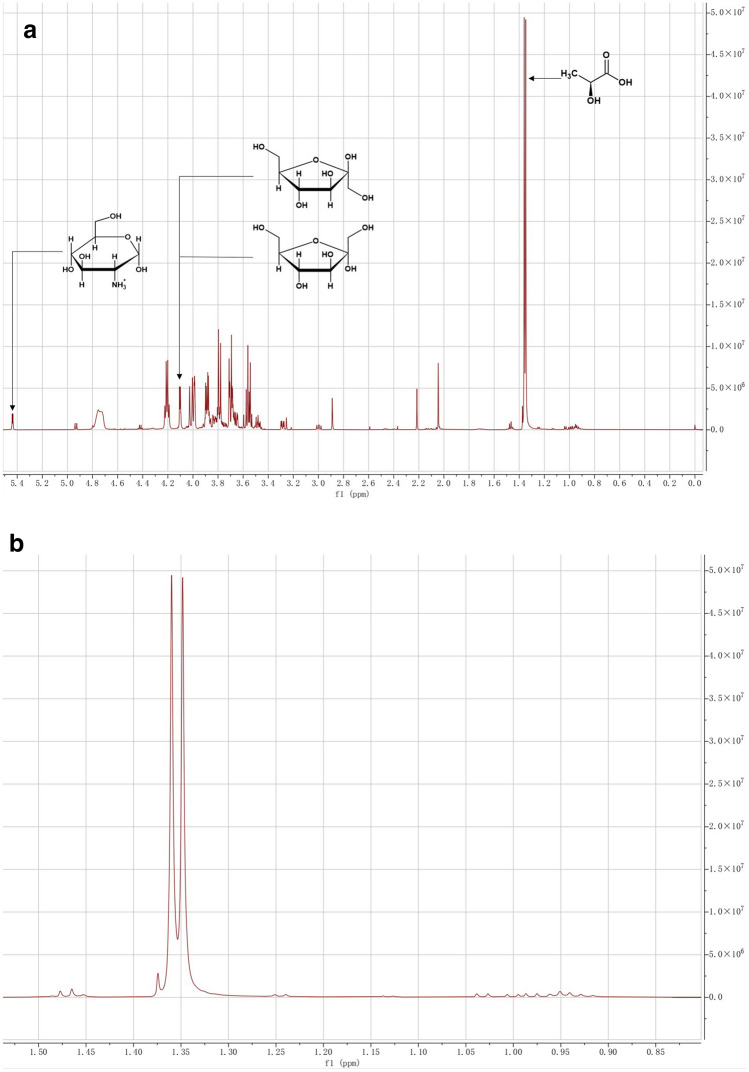
**a** Signal peaks of LA, FT, and glucosamine on 1H NMR Spectrum. **b** The 1H NMR spectrum from 0.85 ppm to 1.50 ppm

The final components in the fermentation broth are represented by the peaks shown in the NMR spectrum (Fig. 1). The signal peak of fructose was observed at 4.12 ppm corresponding to its C3H-β, C4H-β, and C3H-α (Cazor et al. 2006). The signal peak of LA was observed at 1.35 ppm corresponding to its C3H-α. The signal peak of glucosamine was observed at 5.45 ppm corresponding to its C1H-α (Buyondo and Liu 2013). The signal peak of ethanol was not observed at 1.17 ppm (Fig. 1b), which confirmed that the LA fermentation of *L. pentosus* from fructose was homologous.

### Regression model

The quadratic regression models of LA yield and LA productivity are shown as Eq. (5) and Eq. (6), respectively.

5 $$ \begin{aligned} Y_{LA} = & \,0.459432 + 0.003292 \times C_{{{\text{SA}}}} + 0.011139 \times C_{{{\text{PVA}}}} + 0.022315 \times C_{{{\text{CS}}}} \\ & + \,0.002510 \times C_{{{\text{FT}}}} + 0.009396 \times T + 0.039313 \times {\text{pH}} + 0.04167 \times C_{{{\text{SA}}}} \\ & \times \,C_{{{\text{CS}}}} + 0.000035 \times C_{{{\text{FT}}}} \times T + 0.000687 \times T \times {\text{pH}} \\ & - \,0.000635 \times C_{{{\text{SA}}}}^{2} - 0.000833 \times C_{{{\text{PVA}}}}^{2} - 0.023148 \times C_{{{\text{CS}}}}^{2} \\ & - \,0.000016 \times C_{{{\text{FT}}}}^{2} - 0.000234 \times T^{2} - 0.004958 \times {\text{pH}}^{2} \\ \end{aligned} $$

6 $$ \begin{aligned} R_{{{\text{LA}}}} = & \, - \,2.52559 - 0.008323 \times C_{{{\text{SA}}}} + 0.064486 \times C_{{{\text{PVA}}}} + 0.102222 \times C_{{{\text{CS}}}} + 0.024923 \times C_{{{\text{FT}}}} \\ & + \,0.110807 \times T + 0.466958 \times {\text{pH}} + 0.003625 \times C_{{{\text{SA}}}} \times C_{{{\text{PVA}}}} + 0.000227 \times C_{{{\text{SA}}}} \times C_{{{\text{FT}}}} \\ & + \,0.000272 \times C_{{{\text{PVA}}}} \times C_{{{\text{FT}}}} + 0.001222 \times C_{{{\text{CS}}}} \times C_{{{\text{FT}}}} + 0.000137 \times C_{{{\text{FT}}}} \times T - 0.006250 \\ & \times \,C_{{{\text{SA}}}}^{2} - 0.009889 \times C_{{{\text{PVA}}}}^{2} - 0.244444 \times C_{{{\text{CS}}}}^{2} - 0.000152 \times C_{{{\text{FT}}}}^{2} - 0.001727 \times T^{2} \\ & - \,0.038375 \times {\text{pH}}^{2} . \\ \end{aligned} $$

Both regression models are significant with a model p value of less than 0.0001 (Tables 3 and 4). All terms shown in LA yield model are significant with a term p value of smaller than 0.05. In the model of LA production rate, the C_CS_ × C_FT_ term has a minor influence on the result due to a p value of larger than 0.05 but smaller than 0.1, while other terms have significant influence. All these models are well fitted, which is confirmed by the non-significant values of “Lack of Fit”. The high correlation coefficients (*R*^2^) with differences of smaller than 0.2 between adjusted and predicted R^2^ confirms the high accuracy and reasonability of these models. The high adequate precision of larger than 4 and low coefficient of variation confirm the high adequacy and reliability of these models for the prediction of fermentation performance.

**Table 3:** The ANOVA for LA yield of CS film-coated SA-PVA immobilized *L. pentosus* cells

Source	Sum of squares	Degree of freedom	Mean square	*F* value	*p* value
Model	0.0026	15	0.0002	19.98	< 0.0001
C_SA_	0.0002	1	0.0002	26.69	< 0.0001
C_PVA_	0.0002	1	0.0002	23.92	< 0.0001
C_CS_	0.0003	1	0.0003	33.48	< 0.0001
C_FT_	0.0006	1	0.0006	66.06	< 0.0001
T	0.0003	1	0.0003	30.37	< 0.0001
pH	0.0004	1	0.0004	41.30	< 0.0001
C_SA_ × C_CS_	0.0000	1	0.0000	5.69	0.0221
C_FT_ × T	0.0000	1	0.0000	4.11	0.0496
T × pH	0.0001	1	0.0001	6.89	0.0124
C_SA_^2^	0.0001	1	0.0001	7.57	0.0091
C_PVA_^2^	0.0000	1	0.0000	4.12	0.0495
C_CS_^2^	0.0000	1	0.0000	5.08	0.0300
C_FT_^2^	0.0001	1	0.0001	15.75	0.0003
T^2^	0.0001	1	0.0001	16.47	0.0002
pH^2^	0.0003	1	0.0003	28.80	< 0.0001
Residual	0.0003	38	8.782 × 10^–6^		
Lack of Fit	0.0003	33	8.269 × 10^–6^	0.6769*	0.7743
Pure Error	0.0001	5	0.0000		
Cor Total	0.0030	53			
Standard deviation	0.0030		R^2^	0.8875	
Mean	0.9604		Adjusted R^2^	0.8430	
Coefficient of variation (C.V.%)	0.3085		Predicted R^2^	0.7692	
Press	0.0007		Adequate precision	16.4541	

*Non-significant at 5% level

**Table 4:** The ANOVA for LA production rate of CS film-coated SA-PVA immobilized *L. pentosus* cells

Source	Sum of squares	Degree of freedom	Mean square	*F* value	*p* value
Model	0.0563	17	0.0033	48.84	< 0.0001
C_SA_	0.0004	1	0.0004	5.91	0.0202
C_PVA_	0.0013	1	0.0013	18.62	0.0001
C_CS_	0.0004	1	0.0004	6.15	0.0181
C_FT_	0.0025	1	0.0025	36.31	< 0.0001
T	0.0074	1	0.0074	108.98	< 0.0001
pH	0.0010	1	0.0010	14.77	0.0005
C_SA_ × C_PVA_	0.0009	1	0.0009	13.96	0.0006
C_SA_ × C_FT_	0.0007	1	0.0007	10.96	0.0021
C_PVA_ × C_FT_	0.0003	1	0.0003	4.43	0.0424
C_CS_ × C_FT_	0.0002	1	0.0002	3.57	0.0669
C_FT_ × T	0.0005	1	0.0005	8.04	0.0075
C_SA_^2^	0.0064	1	0.0064	94.87	< 0.0001
C_PVA_^2^	0.0051	1	0.0051	75,14	< 0.0001
C_CS_^2^	0.0050	1	0.0050	73.46	< 0.0001
C_FT_^2^	0.0120	1	0.0120	176.76	< 0.0001
T^2^	0.0078	1	0.0078	115.83	< 0.0001
pH^2^	0.0151	1	0.0151	223.53	< 0.0001
Residual	0.0024	36	0.0001		
Lack of Fit	0.0023	31	0.0001	2.36*	0.1713
Pure Error	0.0002	5	0.0000		
Cor Total	0.0587	53			
Standard deviation	0.0082		R^2^	0.9584	
Mean	2.35		Adjusted R^2^	0.9388	
Coefficient of variation (C.V.%)	0.3503		Predicted R^2^	0.8872	
Press	0.0066		Adequate precision	25.4066	

* Non-significant at 5% level

### The interaction of factors on each response

#### The interaction of factors on LA yield

**Fig. 2:**
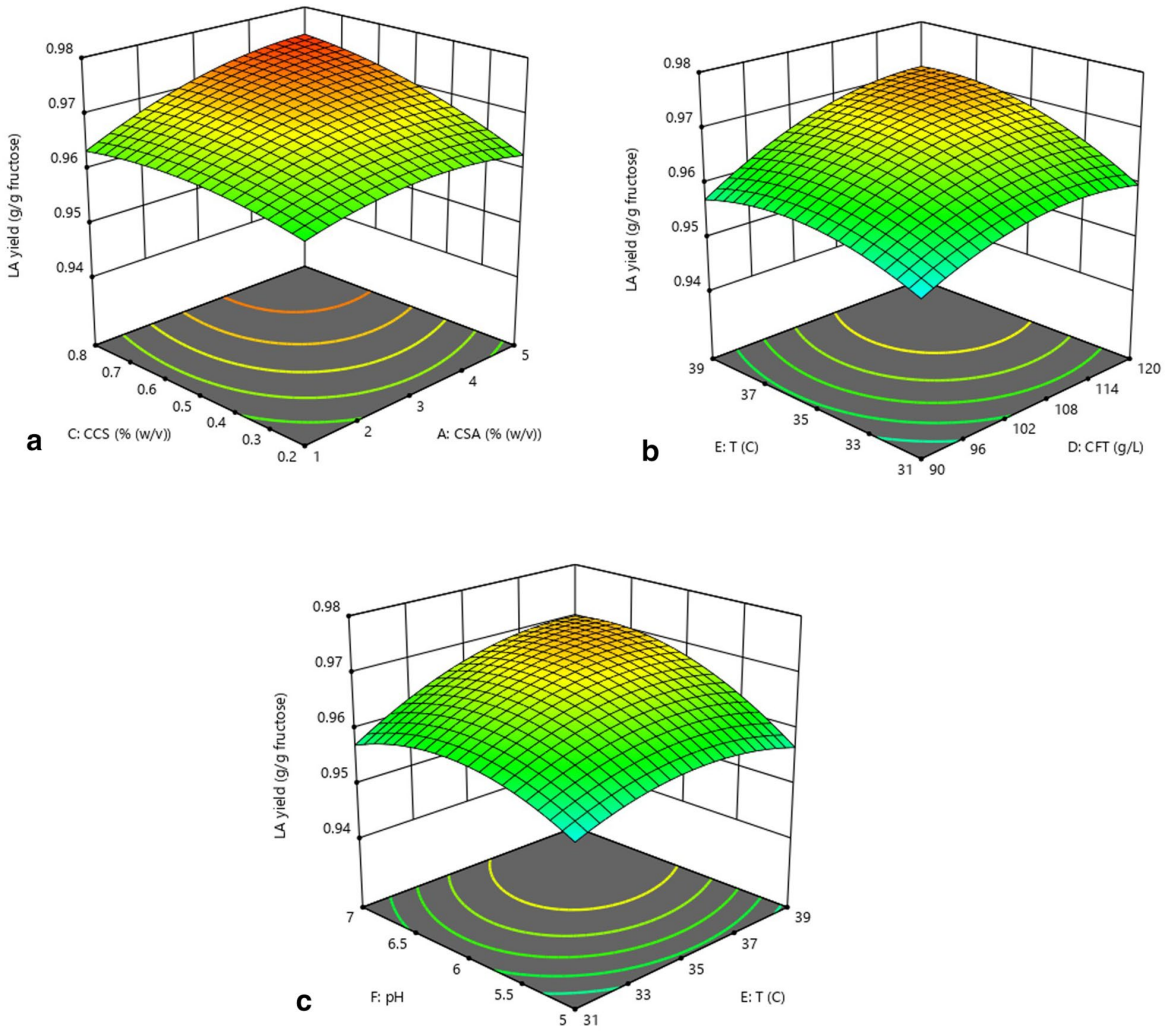
The interaction of **a** SA and CS concentrations, **b** FT concentration and temperature, **c** temperature and pH on LA yield

The LA yield increased significantly with the increase in both SA concentration and CS concentration (Fig. 2a). When the concentration of one gel material is low, the increase in the concentration of the other gel material has no significant effect on the improvement of LA yield. When the CS concentration was low, the cells in the beads could not be effectively encapsulated by the CS film. When the SA concentration was low, the mechanical strength of the beads was reduced, which led to the expansion and breakage of the beads, thus having a negative effect on the encapsulation performance of the beads (Gilson and Thomas 1995). Therefore, a low concentration of SA and CS would cause cell release. More substrate was consumed by these cells for cell growth in a larger space of fermentation medium, which resulted in a decrease in LA yield. High concentrations of SA and CS effectively encapsulated cells in beads, and the cell growth was inhibited, thereby increasing the conversion rate of substrates to LA. Therefore, when the concentration of one gel material is higher, the increase in the concentration of the other gel material can significantly increase the LA yield.

The interaction of temperature and sugar concentration on LA yield is also very significant (Fig. 2b). When the temperature was low, the growth activity of the cells was higher, so more substrate was consumed for cell growth, resulting in low LA yield (Llamas et al. 2020). The increase in the cell content in the beads will also cause further limitations on the efficiency of substrate transport and metabolite synthesis (Wang et al. 2010). Therefore, when the temperature was low, the LA yield could be increased due to a small increase in the FT concentration, but it was not improved significantly with the further increase in FT due to the higher cell density in the beads. As the temperature increases, the LA synthesis activity of cells was gradually enhanced, thereby promoting LA yield. In a suitable temperature range, an increase in the FT concentration resulted in an increase in the net conversion rate of the substrate to LA under cell growth inhibition, which led to a further improvement in LA yield. When the temperature further increased, the metabolic activity of cells was inhibited, and more substrate was consumed for cell maintenance, which caused a reduction in the conversion rate of the substrate to LA, thereby resulting in a decrease in LA yield (Thakur et al. 2018; Sridevi et al. 2015).

The interaction of temperature and pH on LA yield is mainly related to their combined effect on the activity of enzymes that regulate cell growth and metabolism (Hansen et al. 2016). Lower or higher pH will adversely affect LA yield at lower or higher temperatures (Fig. 2c). When one factor is in the suitable range, an appropriate increase in the other factor could significantly improve the LA yield, but its further increase would lead to a decrease in the LA yield.

#### The interaction of factors on LA production rate

**Fig. 3:**
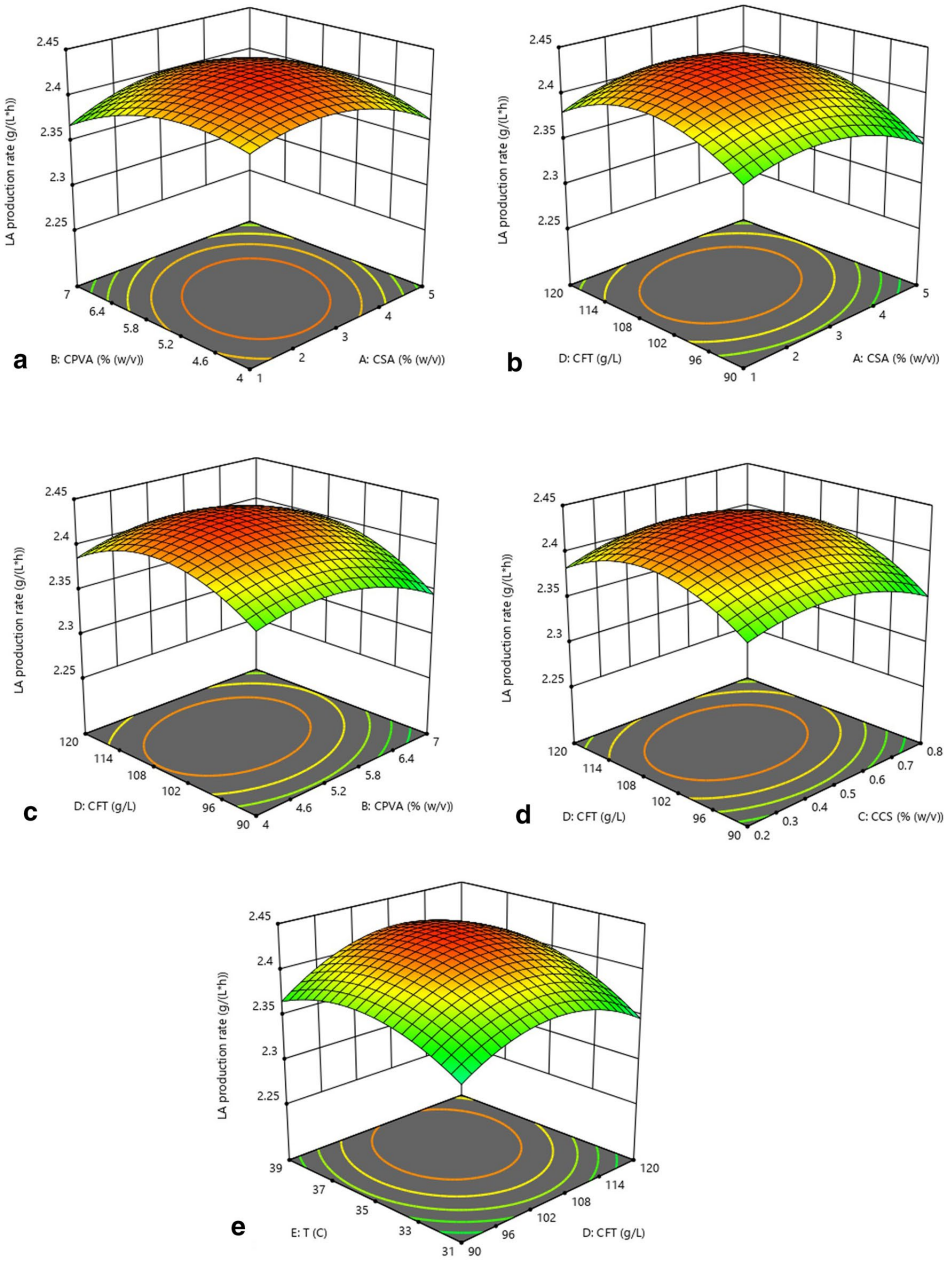
The interaction of **a** SA and PVA concentrations, **b** SA and FT concentrations, **c** PVA and FT concentrations, **d** CS and FT concentrations, **e** FT concentration and temperature on LA production rate

The effects of SA concentration and PVA concentration on LA production rate showed a parabolic behavior (Fig. 3a). As the main gel material for immobilized cell beads, the concentration of SA and PVA has a significant impact on the shape, structure, and mass transfer efficiency of immobilized cell beads. Lower concentrations of SA or PVA would result in a significant decrease in the mechanical strength and surface properties of the beads, which caused shape changes of the beads or even disintegration, thereby resulting in a decrease in fermentation efficiency due to changes in the internal environment of the beads and cell release (Bhatnagar et al. 2016). When the concentration of SA or PVA was higher, the denser structure and reduced surface properties of the beads create greater resistance to the transfer of substrates and nutrients into the bead, thereby negatively affecting the fermentation efficiency (Najafpour et al. 2004). Therefore, the concentrations of both SA and PVA must be controlled within a suitable range to obtain a higher LA production rate.

The influence of the concentration of each gel material and the concentration of FT on the LA production rate also showed a parabolic trend (Fig. 3b–d). The effect of the concentration of each gel material on the LA production rate was still related to the cell viability, mass transfer efficiency, and cell encapsulation performance of the beads affected by the shape, morphology, structural compactness, and surface properties. When the FT concentration is low, cell growth and metabolism were not fully stimulated, which resulted in a decrease in fermentation efficiency due to a lower amount and viability of cells (Bahry et al. 2019). Therefore, an appropriate increase in the FT concentration could increase the difference in FT concentration inside and outside the beads, which would improve the diffusion efficiency of FT into the beads, thereby increasing the LA production rate. When the sugar concentration further increased, the LA production rate decreased slightly, which might be related to the decrease in cell metabolism rate caused by the reduction in water activity in the environment of high FT concentration (Tapia et al. 2020).

The combined effect of temperature and FT concentration on LA production rate was similar to that on LA yield, which was mainly related to the activity of cell growth and metabolism influenced by these two factors. When one factor was at a low level, the promotion of the other factor could not significantly improve the fermentation efficiency (Fig. 3e). When both factors were at a high level, the LA production rate was slightly inhibited, but it could still be maintained at a high level. In addition, when the concentration of the substrate was high, a proper increase in temperature could promote the substrate transport, thereby increasing the utilization and conversion efficiency of the substrate by cells (Vidgren et al. 2010). Therefore, an appropriate increase in both FT concentration and temperature could effectively increase the LA synthesis efficiency of cells.

### Optimization and validation test

By maximizing LA yield and production rate simultaneously, the predicted highest LA yield of 0.971 g/g fructose and highest LA production rate of 2.430 g/(L × h) were obtained at estimated optimal gel material concentrations of 2.809% (w/v) SA, 5.253% (w/v) PVA, and 0.478% (w/v) CS, and estimated fermentation conditions of 107.396 g/L FT, 36.363 °C, and pH 6.084. The validation test was carried out under these optimized conditions. Other conditions are maintained in accordance with previous experiments. The experimental results of LA yield and production rate were obtained as 0.966 ± 0.006 g/g fructose and 2.426 ± 0.018 g/(L × h), respectively. The errors of LA yield and LA production rate were -0.5% and -0.2%, respectively, which confirms the accuracy and reliability of the prediction for fermentation performance and the estimation for optimal conditions. Radosavljević et al. (2020) obtained the highest LA yield of 97.6% and the highest production rate of 0.8 g/(L × h) in the batch fermentation with PVA/Ca-alginate immobilized *L. rhamnosus* cells. Bahry et al. (2019) used the alginate immobilized *L. rhamnosus* cells for LA production from carob pod waste and obtained the highest LA yield and highest production rate of 76.9% and 1.22 g/(L × h), respectively. Thakur et al. (2018) obtained the highest LA yield of 0.921 g/g substrate and the highest LA production rate of 2.28 g/(L × h) in the batch fermentation with SA-CS immobilized *L. casei* cells.

### Fermentation performance of CS film-coated SA-PVA immobilized cells, normal SA-PVA immobilized cells, and free cells

#### Comparison of LA yield and LA production rate of three types of cells at optimized conditions

The conditions for normal SA-PVA immobilized cells and free cells were also optimized based on Box–Behnken design. The normal SA-PVA immobilized cell bead was also prepared by optimized gel material concentrations of 2.203% (w/v) SA and 6.328% (w/v) PVA. The batch fermentation was conducted at the optimal conditions of 112.339 g/L FT, 35.731 °C, and pH 5.914. The batch fermentation of free cells was conducted at 109.167 g/L FT, 34.592 °C, and pH 6.132. The LA yield and production rate of CS film-coated SA-PVA immobilized *L. pentosus* cells were significantly higher than that of no CS film-coated SA-PVA immobilized cells and free cells (Tables 5, 6).

**Table 5:** The comparison of LA production of CS-film-coated SA-PVA immobilized *L. pentosus* cells (SA-PVA-CS) and normal SA-PVA immobilized *L. pentosus* cells (SA-PVA) at 5% level of significance

Cell type	LA yield, g/g fructose	LA production rate, g/(L × h)
SA-PVA-CS	0.967 ± 0.006	2.426 ± 0.018
SA-PVA	0.942 ± 0.008	2.203 ± 0.025
Percentage of increment	2.7%	10.1%
t statistic of increment	4.33	12.54
Degree of freedom	3	3
p value	0.023^	0.001^^^

^^^Significant at 5% level

**Table 6:** The comparison of LA production of CS-film-coated SA-PVA immobilized *L. pentosus* cells (SA-PVA-CS) and free *L. pentosus* cells at 5% level of significance

Cell type	LA yield, g/g fructose	LA production rate, g/(L × h)
SA-PVA-CS	0.967 ± 0.006	2.426 ± 0.018
Free	0.915 ± 0.010	1.637 ± 0.023
Percentage of increment	5.7%	48.4%
t statistic of increment	7.72	46.79
Degree of freedom	3	3
p value	0.002^	< 0.001^^^

^^^Significant at 5% level

### Comparison of LA yield and LA production rate of three types of cells at different temperature

**Fig. 4:**
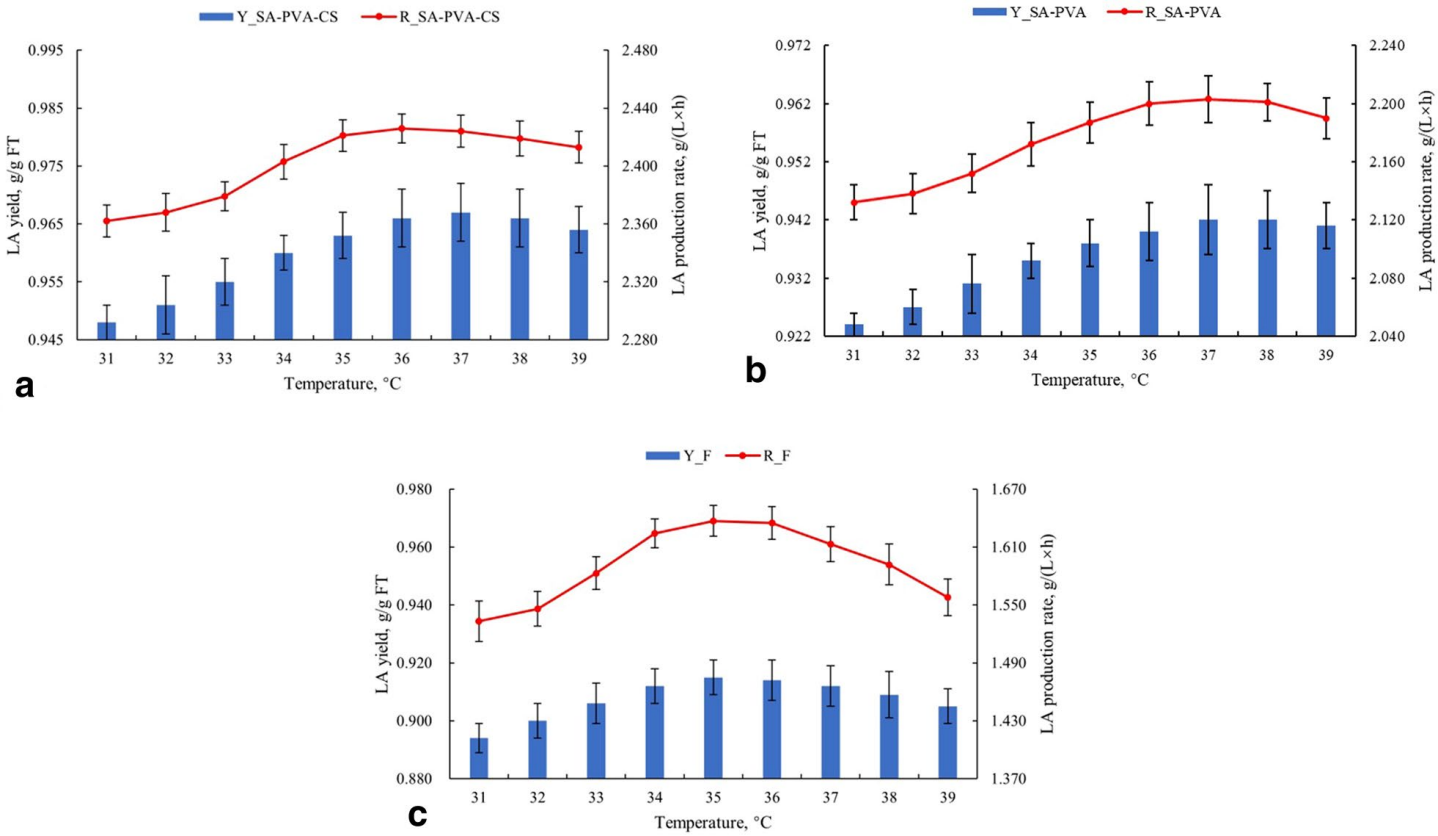
The effect of temperature on LA yield and LA production rate of **a** CS film-coated SA-PVA immobilized cells, **b** normal SA-PVA immobilized cells, and **c** free cells. Other conditions were controlled as the optimal conditions for each cell type

In the selected temperature range, the LA yield and LA production rate of immobilized cells are relatively stable (Fig. 4a, b). When the temperature was lower than the optimal value, the LA yield and LA production rate of immobilized cells increased slowly with the increase in temperature. As the temperature further increased, the LA yield and LA production rate of immobilized cells slightly decreased, but they were all maintained at a high level. However, temperature changes have a more significant effect on the LA yield and LA production rate of free cells (Fig. 4c). This result indicated that the immobilized cells had higher heat stability, but the free cells had a higher heat sensitivity (John et al. 2007). The different trends of LA yield and production rate of two types of immobilized cells in the range of higher temperature were also observed, which indicated that the performance of heat resistance was related to the properties and concentrations of gel materials.

#### Comparison of LA yield and LA production rate of three types of cells at different pH

**Fig. 5:**
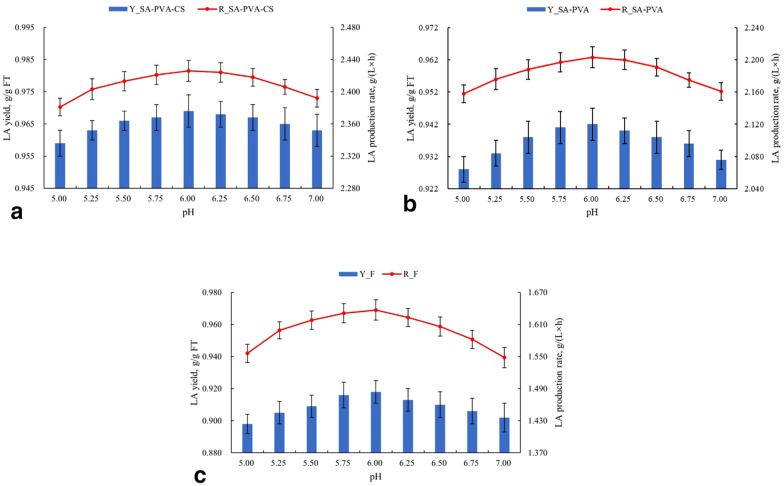
The effect of pH on LA yield and LA production rate of **a** CS film-coated SA-PVA immobilized cells, **b** normal SA-PVA immobilized cells, and **c** free cells. Other conditions were controlled as the optimal conditions for each cell type

The effect of pH on LA yield and LA production rate on three types of cells was similar (Fig. 5a–c). It indicated that the effect of pH was only related to the enzyme activity of cells and the level of product inhibition caused by the undissociated LA (Gonçalves et al. 1997), and it has no relationship with the type and concentration of gel materials. However, free cells still have more significant pH sensitivity.

### Kinetic analysis

For batch fermentation, the cell growth rate was expressed by Eq. (7) based on the Malthus Equation (Liu 2020).

7 $$ r_{X} = \frac{dX}{{dt}} = \mu_{{{\text{Gnet}}}} X = \left( {\mu_{G} - k_{d} } \right)X, $$

where ${r}_{X}$, $X$, $t$, ${\mu }_{\mathrm{Gnet}}$,${\mu }_{G}$, and ${k}_{d}$ represent cell growth rate, cell concentration, fermentation time, net specific cell growth rate, specific cell growth rate, and cell death rate, respectively.

The production rate was expressed by Eq. (8).

8 $$ r_{P} = \frac{dP}{{dt}} = \mu_{P} X, $$

where ${r}_{P}$, $P$, and ${\mu }_{P}$ represent production rate, product (LA) concentration, and specific production rate, respectively.

The substrate (fructose) consumption rate was expressed by Eq. (9) based on the mass balance principles.

9 $$ r_{S} = \frac{dS}{{dt}} = - \left( {\frac{{\mu_{G} }}{{YF_{X/S} }} - \frac{{\mu_{P} }}{{YF_{P/S} }}} \right)X, $$

where ${r}_{S}$, $S$, ${YF}_{X/S}$ and ${YF}_{P/S}$ represent the substrate consumption rate, substrate concentration, yield factor of cell biomass, and yield factor of product, respectively. The yield factors were independent of the substrate concentration (Buyondo and Liu 2013).

For ${\mu }_{G}$, the expression was modified based on the Monod Equation and Verhulst model as Eq. (10) to show a restricted cell growth rate due to the limited nitrogen source, air flow rate. The space for cell growth was also limited for immobilized cells.

10 $$ \mu_{G} = \frac{{\mu_{{{\text{Gmax}}}} S}}{{K_{S} + S}}\left( {1 - \frac{X}{{X_{{{\text{max}}}} }}} \right), $$

where ${\mu }_{Gmax}$, ${K}_{S}$, and ${X}_{max}$ represent maximum cell growth rate, kinetic constant of cell growth, and predicted maximum cell concentration.

Therefore, the kinetic model of ${r}_{X}$ was obtained as Eq. (11).

11 $$ r_{X} = \left[ {\frac{{\mu_{{{\text{Gmax}}}} S}}{{K_{S} + S}}\left( {1 - \frac{X}{{X_{{{\text{max}}}} }}} \right) - k_{d} } \right]X. $$

For ${\mu }_{P}$, the expression was modified based on the Michaelis–Menten equation as Eq. (12) to show an inhibition on LA productivity caused by the combined effects of undissociated and dissociated LA on cytoplasm (Gonçalves et al. 1997).

12 $$ \mu_{P} = \frac{{\mu_{{{\text{Pmax}}}} S}}{{\left( {K_{P} + S} \right)\left( {1 + \frac{P}{{K_{I} }}} \right)}}, $$

where ${\mu }_{\mathrm{pmax}}$ represents maximum production rate. Whereas ${K}_{P}$ and ${K}_{I}$ represent kinetic constant of product synthesis and inhibition, respectively.

Therefore, the kinetic model of ${r}_{P}$ was obtained as Eq. (13).

13 $$ r_{P} = \frac{{\mu_{{{\text{Pmax}}}} S}}{{\left( {K_{P} + S} \right)\left( {1 + \frac{P}{{K_{I} }}} \right)}}X. $$

The expression of ${r}_{s}$ was subsequently obtained as Eq. (14).

14 $$ r_{S} = - \left\{ {\left[ {\frac{{\mu_{{{\text{Gmax}}}} S}}{{K_{S} + S}}\left( {1 - \frac{X}{{X_{{{\text{max}}}} }}} \right)} \right]\frac{1}{{YF_{X/S} }} + \frac{{\mu_{{{\text{Pmax}}}} S}}{{\left( {K_{P} + S} \right)\left( {1 + \frac{P}{{K_{I} }}} \right)YF_{P/S} }}} \right\}X. $$

The kinetic parameters were obtained based on Eqs. (11), (13), (14) (Table 7). Compared to the free cells, the immobilized cells had a lower ${\mu }_{\mathrm{Gmax}}$, which led to a lower rate of cell growth than that of free cells. Although the growth of immobilized cells is further inhibited compared to free cells, the substrate is still consumed at a high rate for LA synthesis. Therefore, when both cell growth and LA synthesis are involved, the ${K}_{S}$ of immobilized cells is still much smaller than that of free cells. The lower ${X}_{\mathrm{max}}$ of immobilized cells also reflected the growth inhibition of cells. The immobilized cells also had higher ${\mu }_{\mathrm{Pmax}}$ and lower ${K}_{P}$, which indicated that the immobilized cells had a higher metabolic activity, and it takes less time to reach the maximum metabolic rate, resulting in a higher overall rate of LA synthesis. The ${K}_{I}$ values of the three types of cells were similar, which indicated that the levels of product inhibition were similar due to their close values of optimized pH. The higher ${YF}_{P/S}$ and lower ${YF}_{X/S}$ of immobilized cells indicated a higher efficiency of substrate conversion to LA. The ${k}_{d}$ of immobilized cells was negligible compared to that of free cells, which also indicated higher cell viability of immobilized cells. The better fermentation performance of CS film-coated SA-PVA immobilized cells compared to that of normal SA-PVA immobilized cells mainly resulted from the higher overall cell viability due to the better performance of cell encapsulation. In Eqs. (11), (13), and (14), the influence of the substrate concentration on the rates of cell growth and LA synthesis can be intuitively reflected. By continuous integration, the concentrations of cell and LA at any time point in the fermentation period can be obtained simultaneously.

**Table 7:** Kinetic parameters of batch fermentation by CS film-coated SA-PVA immobilized cells (SA-PVA-CS), normal SA-PVA immobilized cells (SA-PVA), and free cells (Free)

Parameter	Unit	Value		
		SA-PVA-CS	SA-PVA	Free
${\mu }_{G\mathrm{max}}$	h^−1^	0.1187	0.1420	0.2732
${K}_{S}$	g/L	0.2842	0.1834	3.1813
${X}_{\mathrm{max}}$	g/L	3.1321	5.6815	10.7424
${\mu }_{P\mathrm{max}}$	h^−1^	187.2759	127.1298	53.1298
${K}_{P}$	g/L	28.2213	50.0217	61.8285
${K}_{I}$	g/L	0.7534	0.7161	0.7843
${YF}_{P/S}$	g/g	1.8193	1.4653	1.2958
${YF}_{X/S}$	g/g	0.0327	0.1195	0.4893
${k}_{d}$	h^−1^	–	–	0.0063

**Fig. 6:**
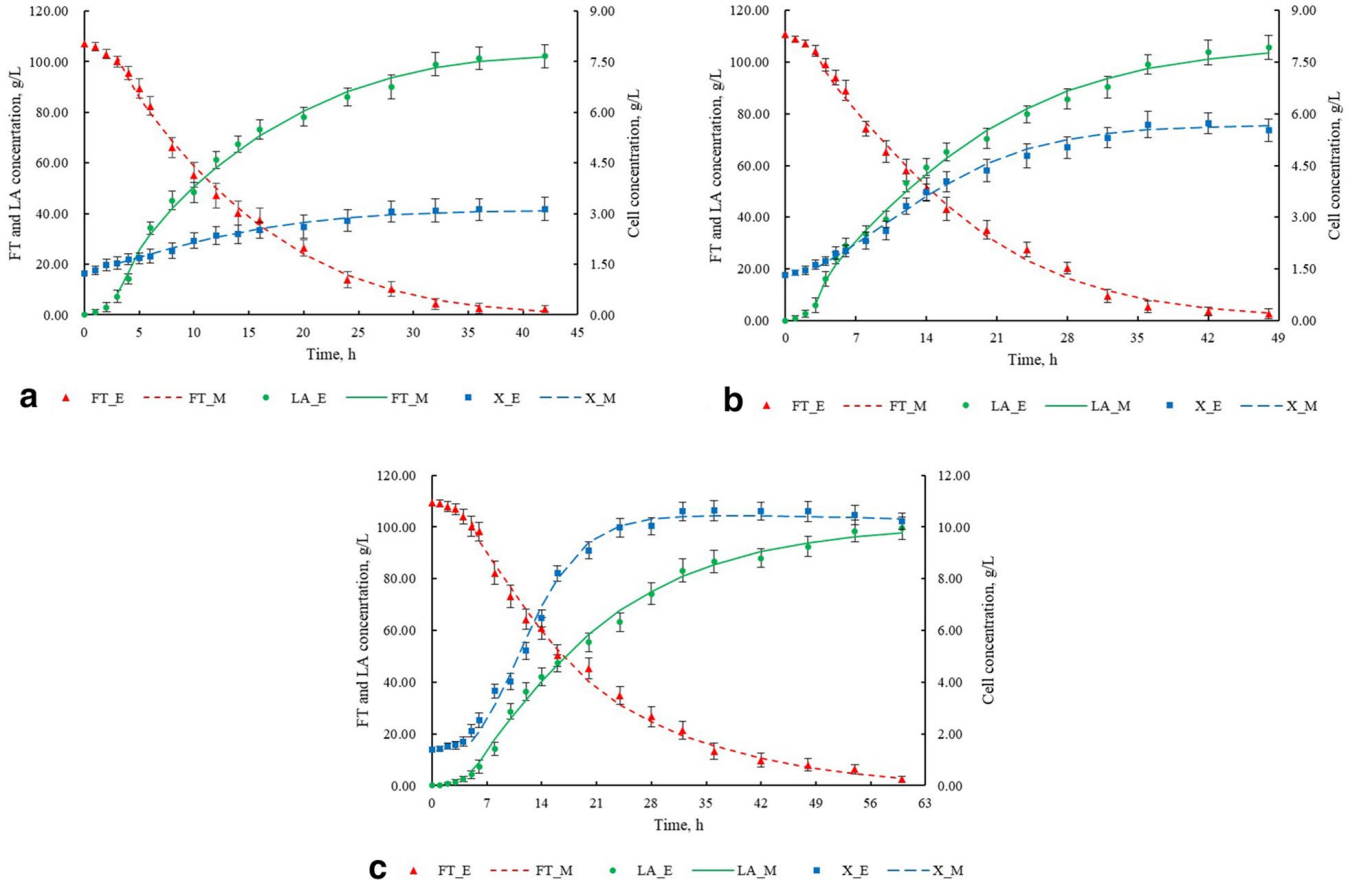
The experimental results and model prediction results of **a** CS film-coated SA-PVA immobilized cells, **b** normal SA-PVA immobilized cells, and **c** free cells. F_E, LA_E, and X_E represent the experimental data of FT concentration, LA concentration, and cell concentration, respectively. F_M, LA_M, and X_M represent the model-predicted data of FT concentration, LA concentration, and cell concentration, respectively

The fermentation periods of CS film-coated SA-PVA immobilized cells, normal SA-PVA immobilized cells, and free cells were 42 h, 48 h, and 60 h, respectively (Fig. 6a–c). The fermentation performance of the three cell types mainly depends on their growth and metabolic activity. Immobilized cells have lower growth activity but higher metabolism levels than free cells. Compared with normal SA-PVA immobilized cells, the application of CS film further inhibited the activity of cell growth and improved efficiency of cell metabolism, which were also reflected by the kinetic parameters of two types of immobilized cells. The kinetic models of fructose consumption, LA synthesis, and cell growth have high correlation coefficients (Table 8). These models can be used to mathematically predict the fermentation performance of three cell types from fructose with high accuracy and reasonability.

**Table 8:** Correlation coefficients (R^2^) between the experimental results and model prediction results of CS film-coated SA-PVA immobilized cells (SA-PVA-CS), normal SA-PVA immobilized cells (SA-PVA), and free cells (Free)

Cell type	FT concentration	LA concentration	Cell concentration
SA-PVA-CS	0.9868	0.9955	0.9939
SA-PVA	0.9920	0.9949	0.9930
Free	0.9909	0.9935	0.9942

### Repeated batch fermentation

The CS film-coated SA-PVA immobilized cells could maintain high LA yield and production rate in nine batches, which implied these immobilized cells had good reusability (Table 9). The significant decrease in LA yield and production rate started from the 10^th^ batch, and the breakage of the beads was observed in the 13th batch. The normal SA-PVA immobilized cells could maintain a high LA yield and production rate in ten batches, and the breakage of the beads was observed in the 15th batch. It could be confirmed that both CS film-coated SA-PVA immobilized cells and normal SA-PVA immobilized cells have excellent mechanical stability to avoid the bead breakage due to the cell growth and continuous stirring. Compared with normal SA-PVA immobilized cells, the slightly lower mechanical strength of CS film-coated SA-PVA immobilized cells might be due to the lower optimal PVA concentration. A higher concentration of PVA can effectively prevent the reduction in mechanical strength caused by the bead expansion in the repeated batch fermentation process. However, the LA yield and production rate of CS film-coated SA-PVA immobilized cells in each batch was always much higher than that of normal SA-PVA immobilized cells.

**Table 9:** Performance of repeated batch fermentation by CS film-coated SA-PVA immobilized cells (SA-PVA-CS) and normal SA-PVA immobilized cells (SA-PVA)

Batch	SA-PVA-CS		SA-PVA	
	LA yield Y_LA_, g/g FT	LA production rate R_LA_, g/(L × h)	LA yield Y_LA_, g/g FT	LA production rate R_LA_, g/(L × h)
1	0.967 ± 0.009	2.426 ± 0.018	0.942 ± 0.008	2.203 ± 0.025
2	0.971 ± 0.006	2.431 ± 0.017	0.946 ± 0.007	2.207 ± 0.021
3	0.960 ± 0.011	2.415 ± 0.021	0.947 ± 0.011	2.210 ± 0.019
4	0.964 ± 0.008	2.417 ± 0.021	0.949 ± 0.012	2.212 ± 0.021
5	0.966 ± 0.007	2.422 ± 0.022	0.946 ± 0.008	2.208 ± 0.018
6	0.973 ± 0.005	2.427 ± 0.019	0.943 ± 0.006	2.207 ± 0.017
7	0.963 ± 0.010	2.423 ± 0.021	0.941 ± 0.009	2.203 ± 0.019
8	0.962 ± 0.009	2.411 ± 0.021	0.941 ± 0.007	2.206 ± 0.023
9	0.959 ± 0.006	2.409 ± 0.023	0.946 ± 0.008	2.206 ± 0.024
10	0.953 ± 0.007	2.401 ± 0.026	0.943 ± 0.010	2.201 ± 0.018
11	0.947 ± 0.008	2.394 ± 0.022	0.938 ± 0.012	2.194 ± 0.019
12	0.942 ± 0.009	2.375 ± 0.025	0.934 ± 0.001	2.182 ± 0.024
13	0.939 ± 0.005	2.361 ± 0.024	0.927 ± 0.008	2.169 ± 0.026
14	–	–	0.921 ± 0.009	2.157 ± 0.022
15	–	–	0.913 ± 0.006	2.146 ± 0.023

### Conclusion

Under the optimal conditions, the highest LA yield and production rate of CS film-coated SA-PVA immobilized cells can be obtained as 0.966 ± 0.006 g/g fructose and 2.426 ± 0.018 g/(L × h), respectively, which have better fermentation performance than that of normal SA-PVA immobilized cells and free cells. The errors of LA yield and LA production rate were − 0.5% and − 0.2, respectively, which confirms the accuracy and reliability of the prediction for fermentation performance and the estimation for optimal conditions. The immobilized cells have excellent heat stability, while the free cells have higher sensitivity to temperature and pH. The kinetic parameters can effectively describe the fermentation performance of different types of cells. The kinetic models of CS film-coated SA-PVA immobilized cells can be used to describe the tendency of fructose consumption, LA production, and cell growth during the complete fermentation period. The CS film-coated SA-PVA immobilized cells with excellent mechanical strength have good reusability for repeated batch fermentation.

## Data Availability

All data obtained or analyzed during this study are included in this article and available from the corresponding author.

## Abbreviations

ANOVA: Analysis of variance
ATCC: American Type Culture Collection
CS: Chitosan
EMP: Embden–Meyerhoff–Parnas
FT: Fructose
LA: Lactic acid
LAB: Lactic acid bacteria
MRS: De Man, Rogosa and Sharpe
1H NMR: Proton nuclear magnetic resonance
OD: Optical density
PK: Pentose phosphoketolase
PLA: Polylactic acid
PVA: Polyvinyl alcohol
RSM: Response surface methodology
SA: Sodium alginate

## Annotation of kinetic parameters

μ_Gmax_: Maximum cell growth rate
K_S_: Kinetic constant of cell growth
X_max_: Predicted maximum cell concentration
μ_Pmax_: Maximum production rate
K_P_: Kinetic constant of product synthesis
K_I_: Kinetic constant of product inhibition
YF_P/S_: Yield factor of cell biomass
YF_X/S_: Yield factor of product
K_d_: Cell death rate
